# Supplementary material for: Requirement for p62 acetylation in the aggregation of ubiquitylated proteins under nutrient stress
Source: Nat Commun. 2019 Dec 19;10:5792. doi: 10.1038/s41467-019-13718-w (PMC6923396; doi:10.1038/s41467-019-13718-w)
Supplement: Supplementary file 1 — Supplementary Information [file 41467_2019_13718_MOESM1_ESM.pdf]

Supplementary Information

Requirement for p62 acetylation in the aggregation of ubiquitylated proteins under nutrient stress

You et al.

Supplementary Figures

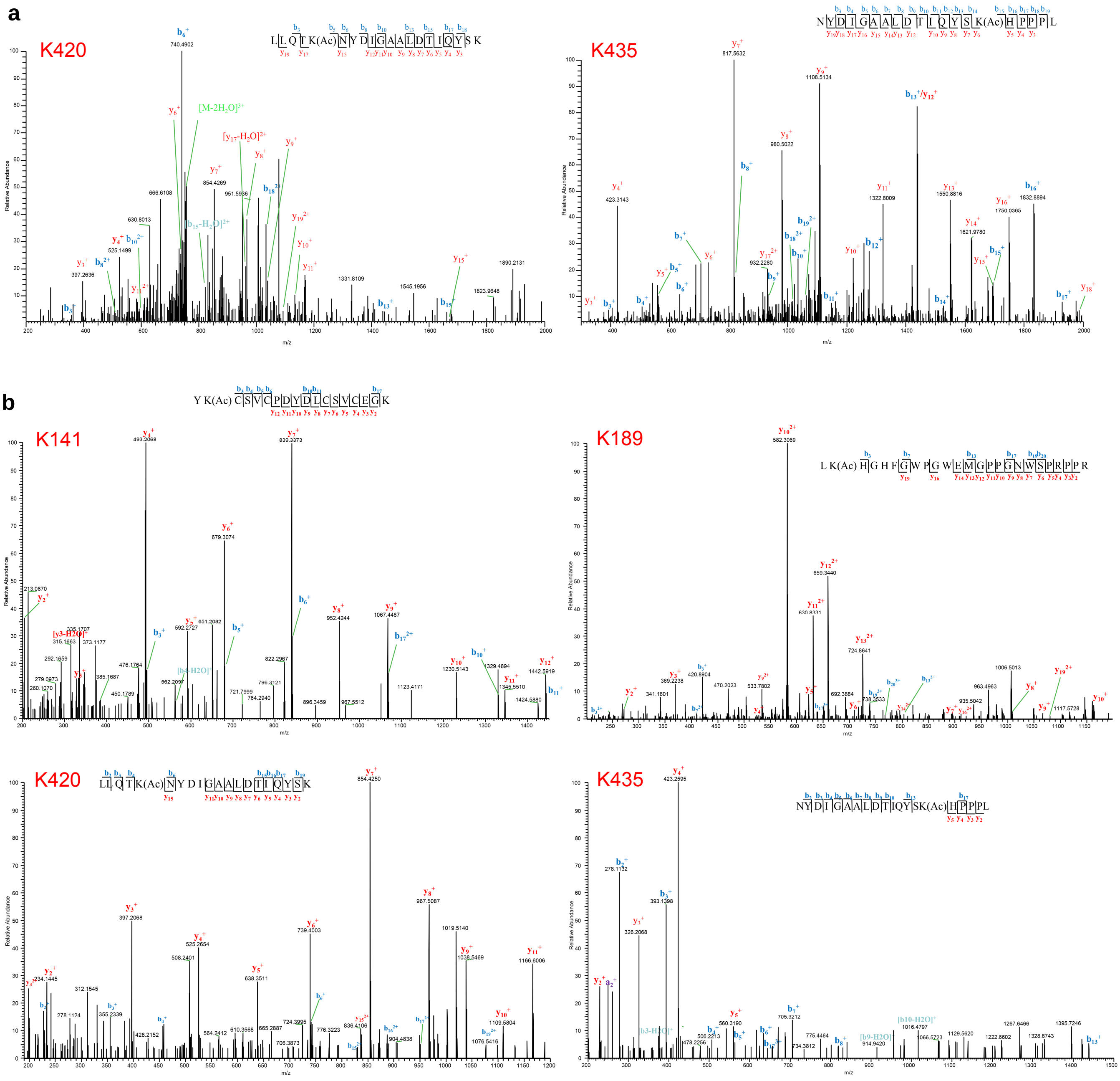

Supplementary Figure 1

MS/MS spectra of tryptic peptides (inset) of purified recombinant p62 protein after in vitro acetylation (**a**) or Flag-p62 immunoprecipitated from TSA-treated Flag-p62-HEK293T cells (**b**) showing a mass shift of 42.01 Da at the lysine residues.

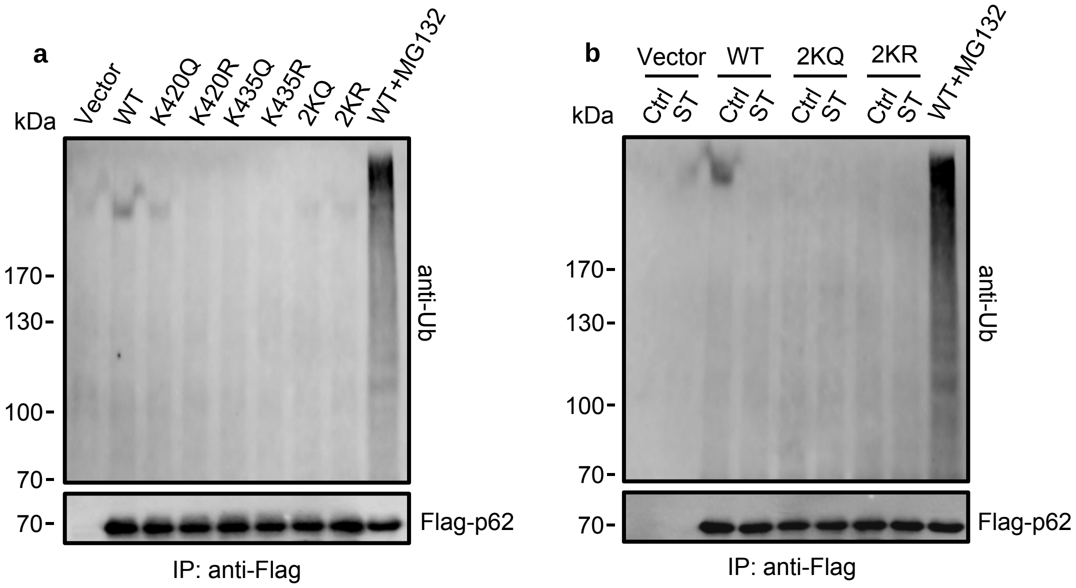

Supplementary Figure 2 p62 acetylation and cell starvation do not cause the ubiquitylation of p62.

(**a**,**b**) *p62*-KO HEK293 cells transiently expressing Flag-tagged WT p62 or each of the indicated p62 mutants were lysed with urea buffer. Then the Flag-p62 proteins were immunoprecipitated with anti-Flag and the precipitates were analyzed using anti-Ub. Source data are provided as a Source Data file.

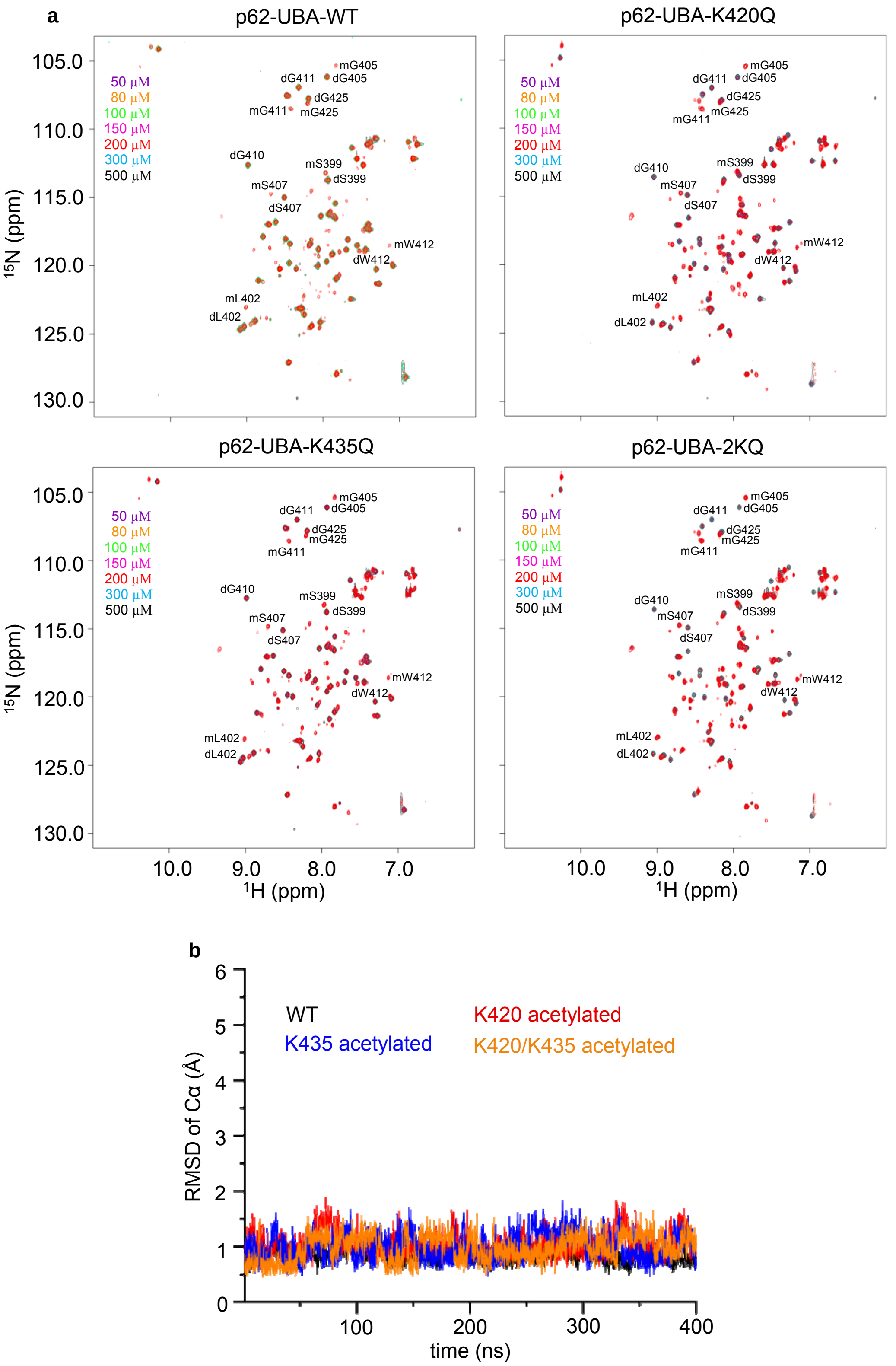

Supplementary Figure 3 Acetylation at K420 and K435 disrupts UBA dimerization.

(**a**) ^{1}H-^{15}N correlation spectra of each of the indicated p62-UBA proteins. Amino acid residues

used to fit the K_D value are denoted. d: dimer; m: monomer. (**b**) The trajectories for each UBA subunit from the MD simulations for WT, K420 acetylated, K435 acetylated and K420/K435 acetylated p62-UBA dimers. The fluctuations in Cα RMSD are plotted over a time window of 400 ns. Source data are provided as a Source Data file.

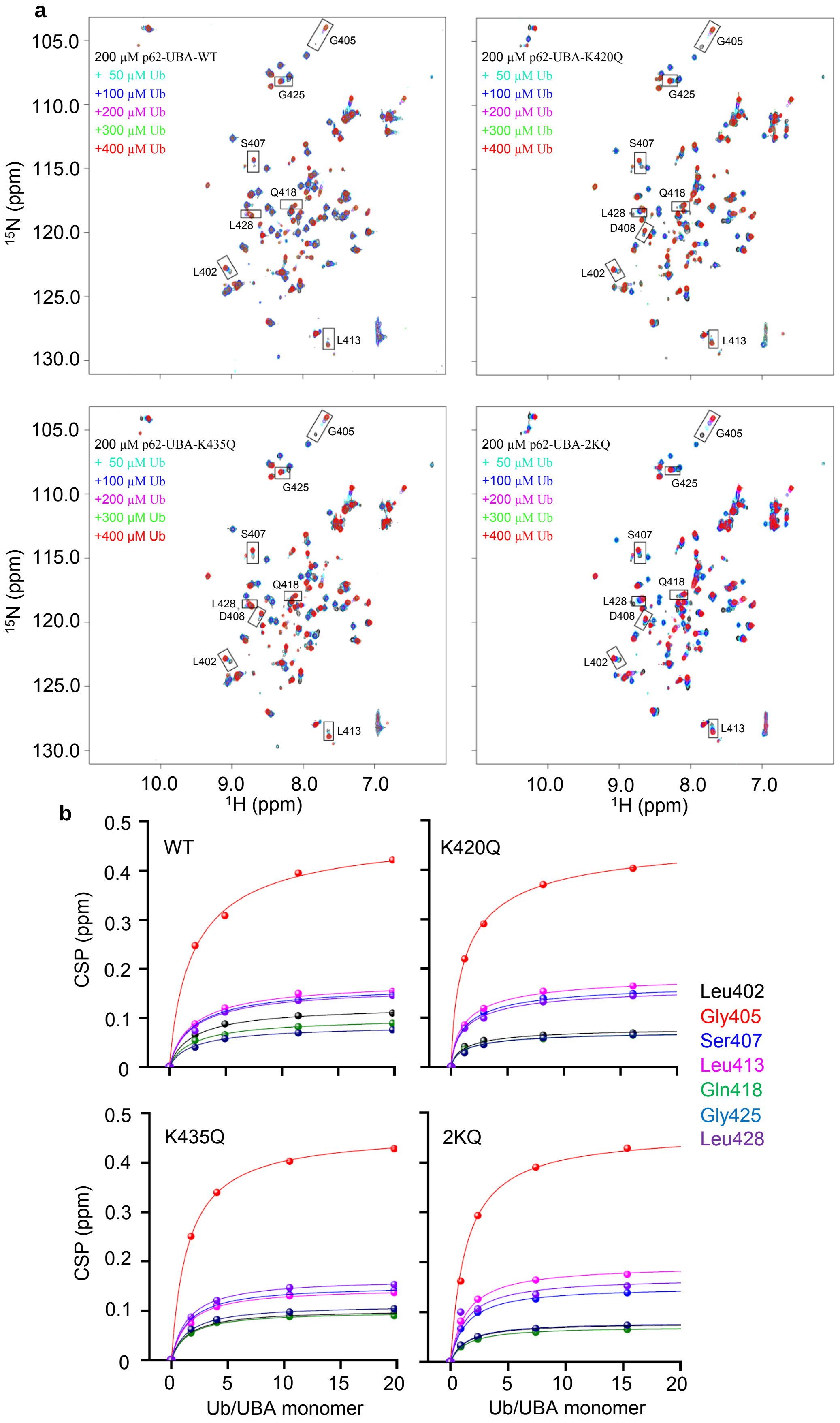
a
200 μM p62-UBA-WT
+ 50 μM Ub
+100 μM Ub
+200 μM Ub
+300 μM Ub
+400 μM Ub
200 μM p62-UBA-K420Q
200 μM p62-UBA-K435Q
200 μM p62-UBA-2KQ
G405
G425
S407
Q418
L428
D408
L402
L413
15N (ppm)
1H (ppm)
b
WT
K420Q
K435Q
2KQ
CSP (ppm)
Ub/UBA monomer
Leu402
Gly405
Ser407
Leu413
Gln418
Gly425
Leu428

Supplementary Figure 4 K435 acetylation directly enhances the p62-ubiquitin binding.

(**a**) ^{1}H-^{15}N correlation spectra of p62-UBA in the presence of increasing concentrations of ubiquitin. Amino acid residues used to fit the dissociation constant are marked. (**b**) Changes of chemical shift values over ubiquitin concentrations for the binding-perturbed residues were globally fitted to a standard 1:1 binding equation. The chemical shift differences are expressed as $[0.5 \times (\Delta\delta H^2 + 0.2 \times \Delta\delta N^2)]^{0.5}$, in which $\Delta\delta H$ and $\Delta\delta N$ are the chemical shift perturbations (CSP) in ppm unit in the proton and nitrogen dimensions, respectively. Note that the ubiquitin binding equilibrium is affected by the amount of available p62-UBA monomer. Source data are provided as a Source Data file.

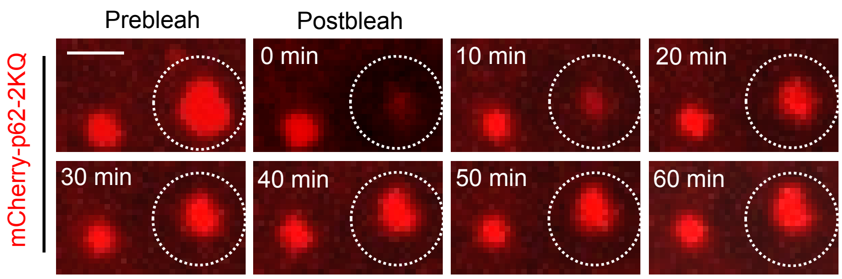

Supplementary Figure 5 Acetylation enhances p62 body formation.

FRAP analysis of mCherry-p62-2KQ clusters. A representative time-course is shown. The experiments were performed 10 min after mixing mCherry-p62-2KQ (5 μM) and 8×ubiquitin (1.5 μM) at RT. Scale bar, 2 μm.

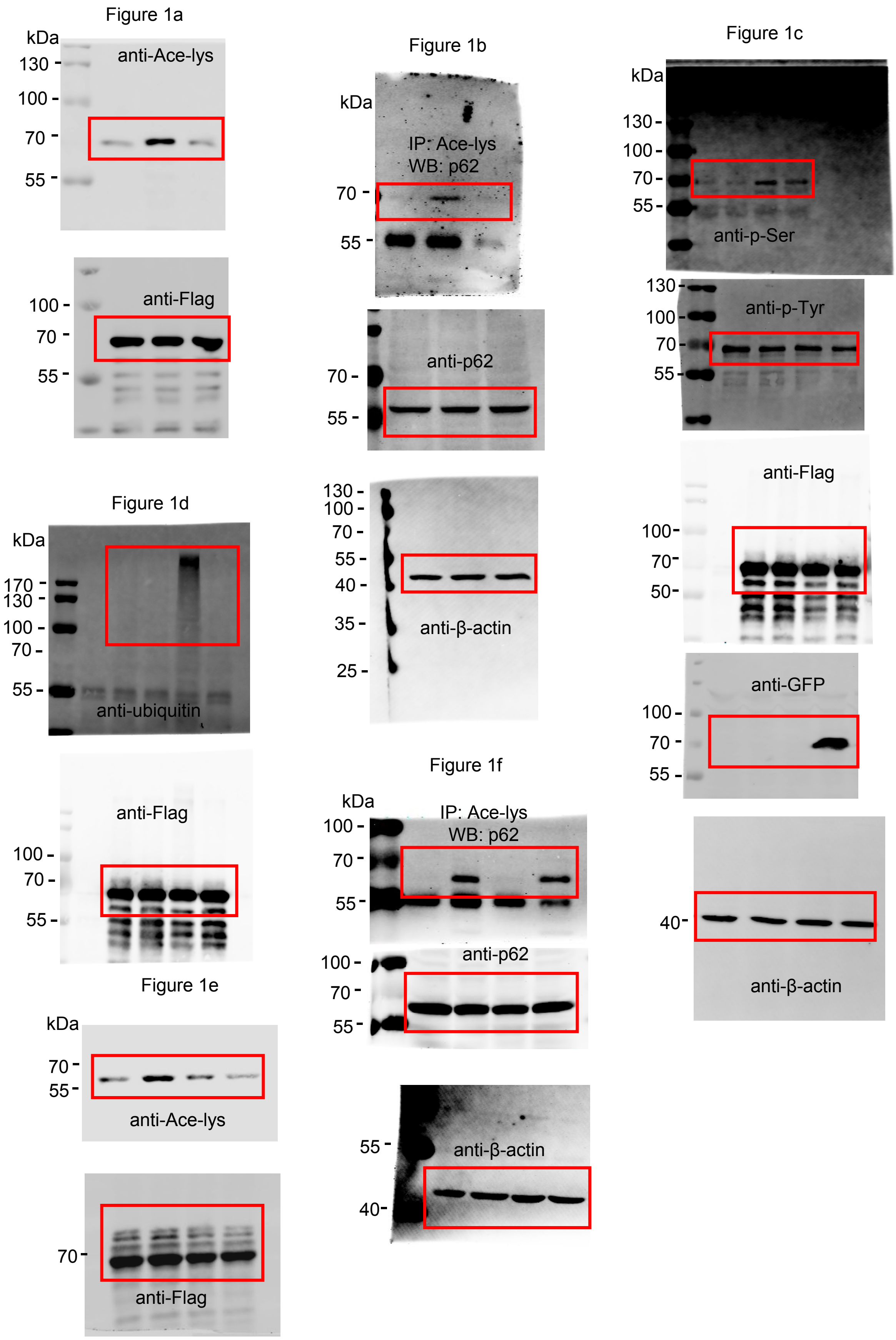

Supplementary Figure 6 Uncropped blots/gels

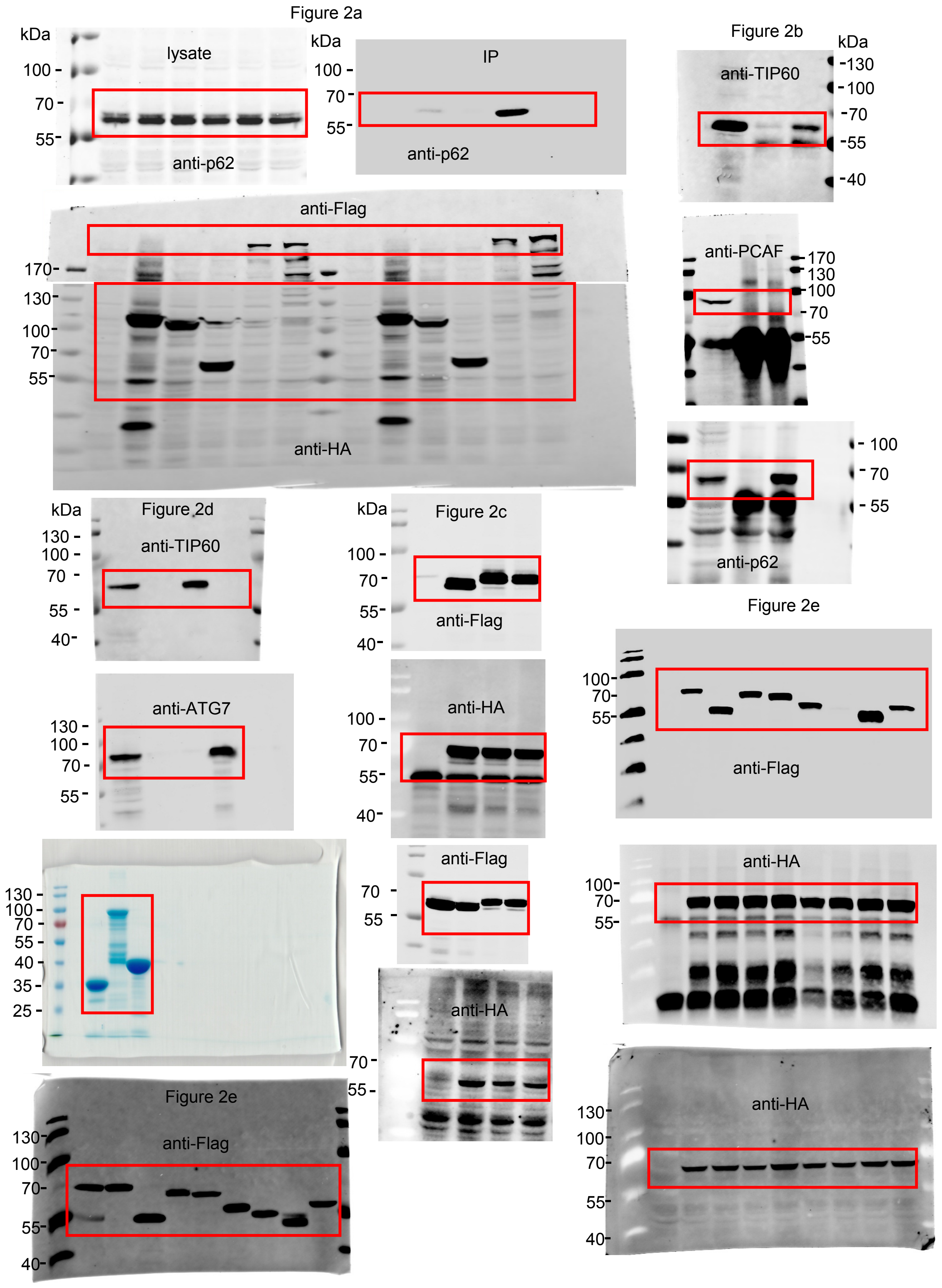

Supplementary Figure 7 Uncropped blots/gels

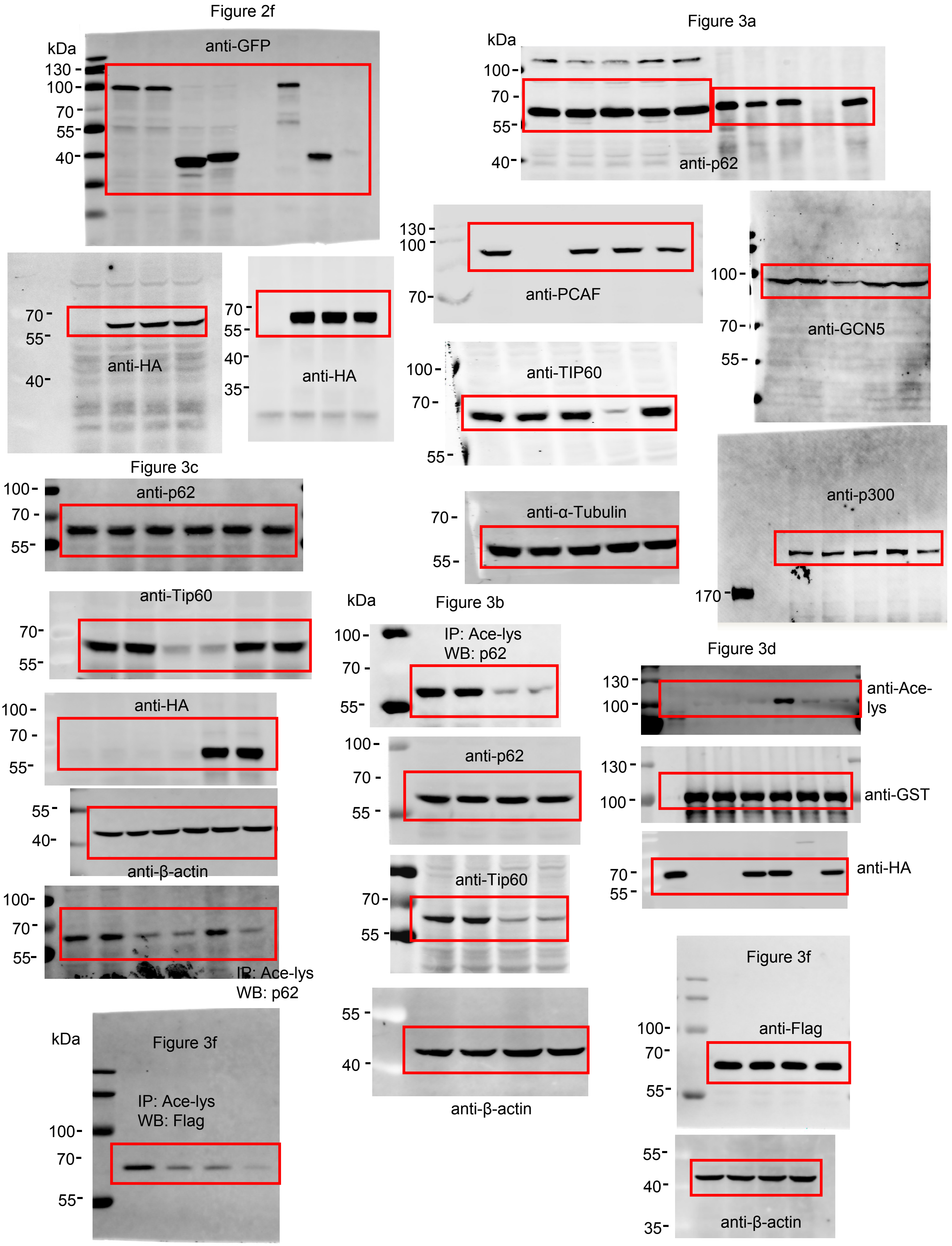

Supplementary Figure 8 Uncropped blots/gels

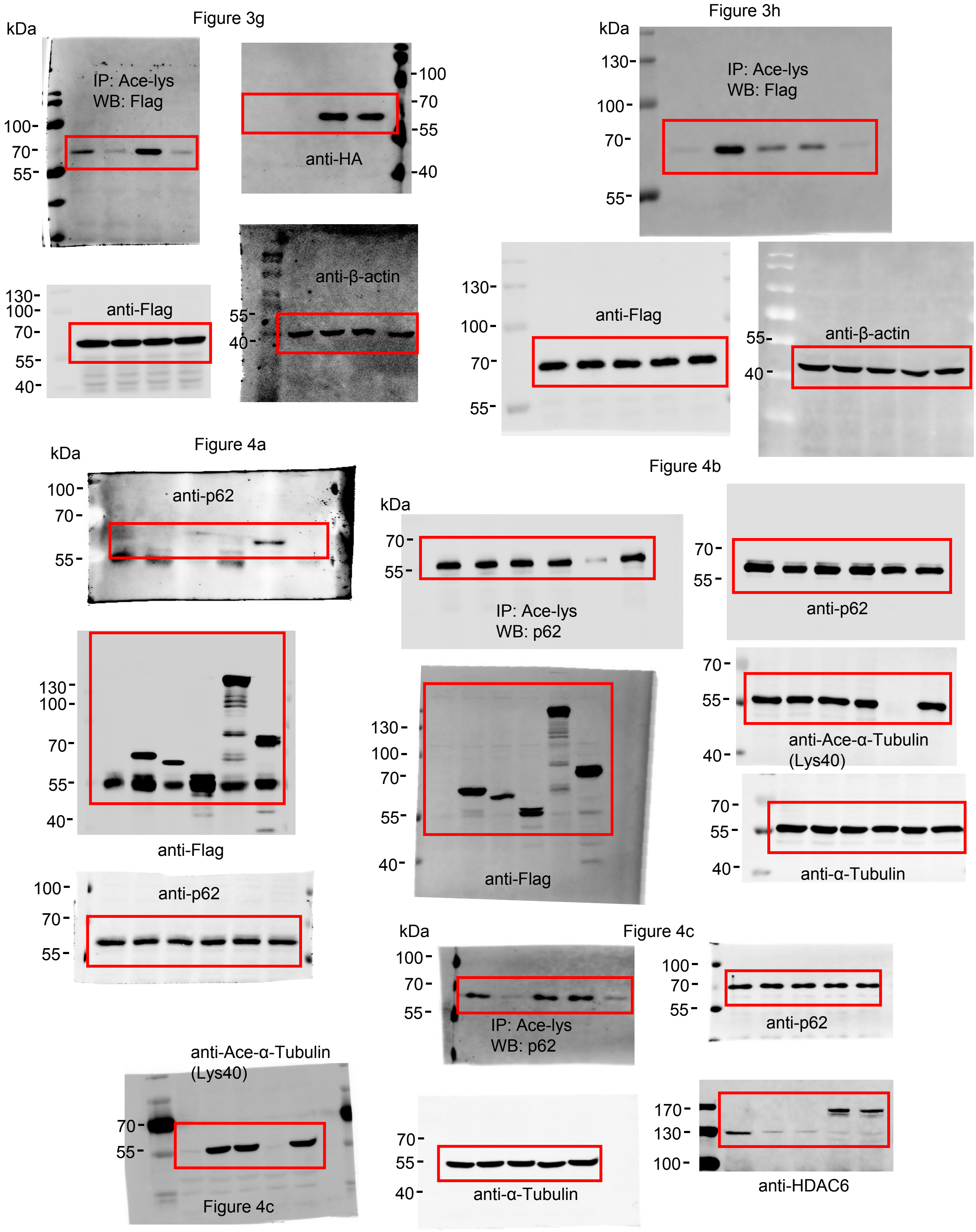

Supplementary Figure 9 Uncropped blots/gels

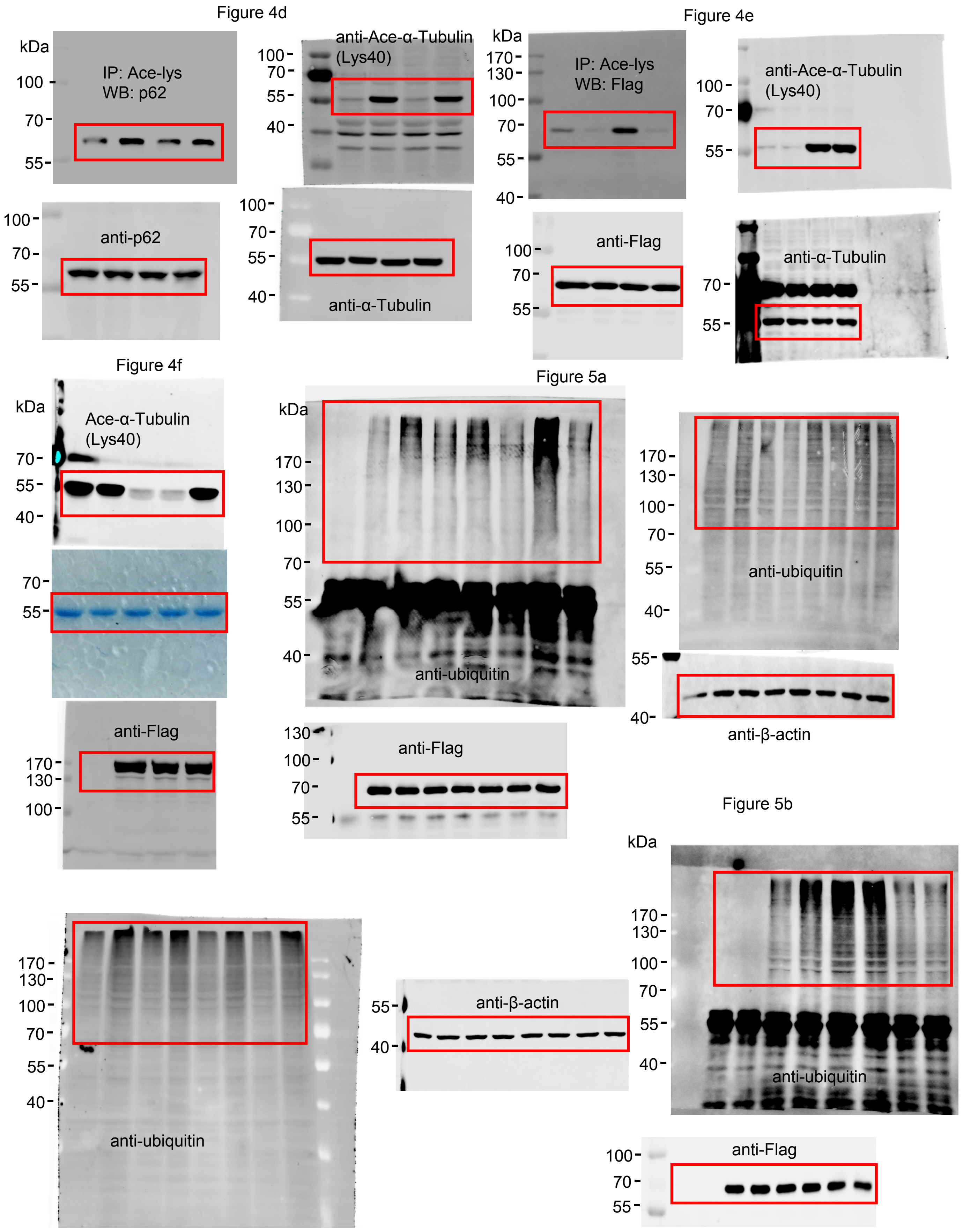

Supplementary Figure 10 Uncropped blots/gels

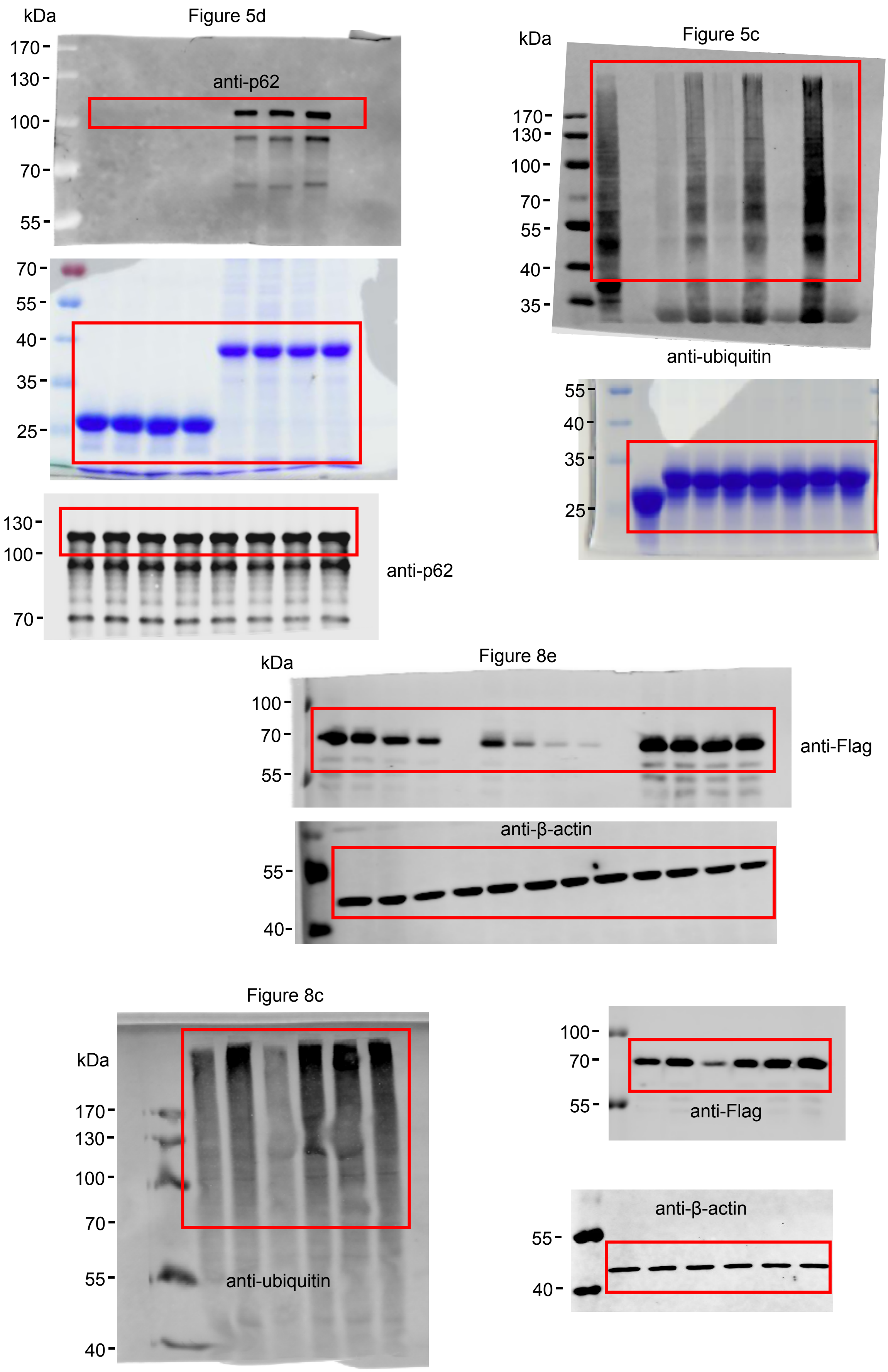

Supplementary Figure 11 Uncropped blots/gels

Supplementary Figure 2a

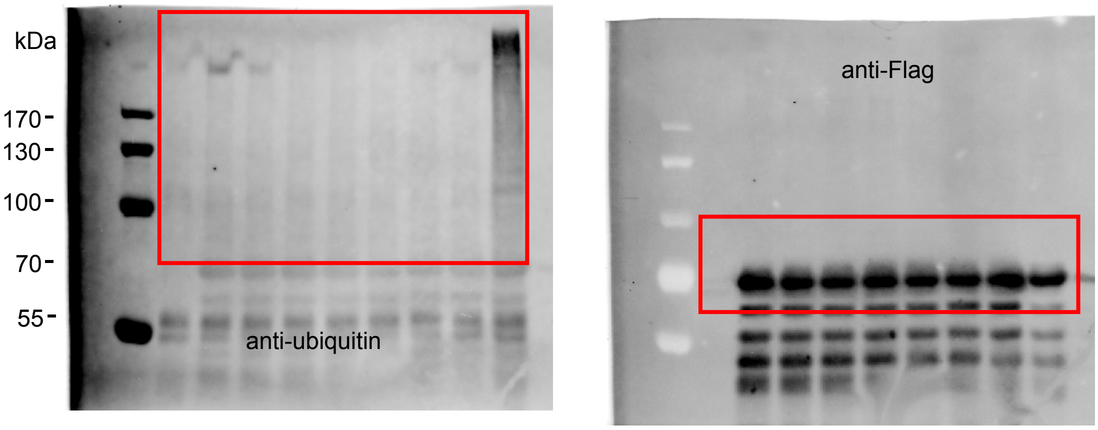

Supplementary Figure 2b

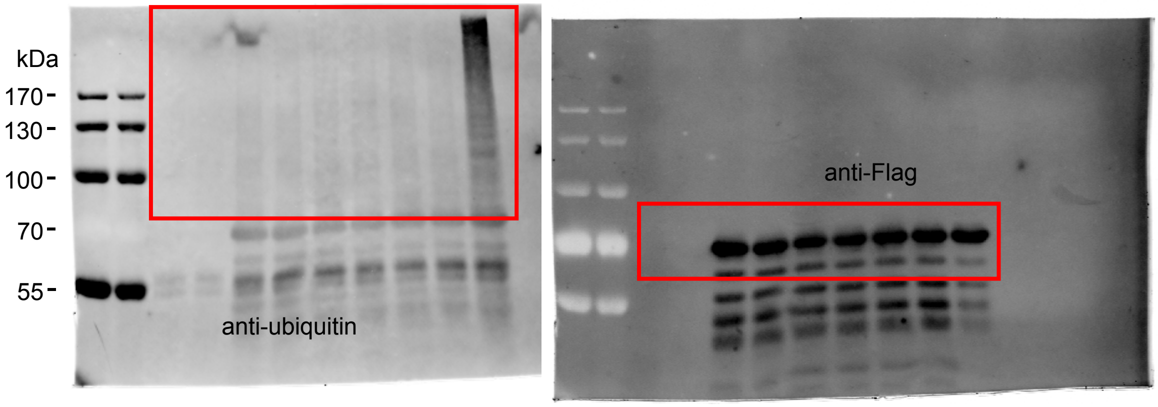

Supplementary Figure 12 Uncropped blots/gels

Supplementary Tables

Supplementary Table 1 List of primers used in this study.

Primers	Sequences (5'-3')
Flag/GST-p62-F-EcoRI	CCGGAATTCATGGCGTCGTTCACGGTG
Flag/GST-p62-R-XhoI	CCGCTCGAGTCACAATGGTGGAGGGTGC
mCherry-p62-F-XhoI	CCGCTCGAGCTATGGCGTCGTTCACGGTG
mCherry-p62-R-EcoRI	CCGGAATTCTCACAATGGTGGAGGGTGC
GST-mCherry-p62-F-EcoRI	CGGGAATTCATGGTGAGCAAGGGCGAGGAG
GST-mCherry-p62-R-NotI	AAGGAAAAAAGCGGCCGCTCTCACAATGGTGG
GST-8×ubiquitin-F-BamHI	CGCGGATCCATGCAGATCTTCGTG
GST-8×ubiquitin-R-NotI	AAGGAAAAAAGCGGCCGCTTACCCACCTCT
6×His-ubiquitin-F-EcoRI	CCGGAATTCATGCAGATCTTCGTG
6×His-ubiquitin-R-XhoI	CCGCTCGAGTTACCCACCTCTGAG
p62-Δ1-85-F	GCTATGTCCTATGTGAAAG
p62-Δ1-85-R	GAATTCCTTGTCATCGTCATC
p62-Δ86-121-F	GTGCACCCCAATGTGATCTG
p62-Δ86-121-R	CATTGTCAGCTCCTCATCAC
p62-Δ122-168-F	TTTCCCAACCCCTTTG
p62-Δ122-168-R	CATGTTTCGGGGTGCC
p62-Δ169-253-F	GAGGTTGACATTGATGTGG
p62-Δ169-253-R	GATGAGCTTGCTGTGTTC
p62-Δ254-310-F	GAGCAAATGAAAAAGATAG
p62-Δ254-310-R	AATGCCTAGAGGGCTGAG
p62-Δ311-384-F	GCCCTATACCCACATCTC
p62-Δ311-384-R	TGTCAGAGACTGAGCAGG
p62-Δ385-440-F	TGACTCGAGGGCCGCATAGA
p62-Δ385-440-R	AGCTTCCTTCAGCCCTGTG
p62-254-310-XhoI-F	CCGCTCGAGCTGAGGTTGACATT
p62-254-310-EcoRI-R	CCGGAATTCTCATGTCAGAGACTG
p62-311-384-XhoI-F	CCGCTCGAGCTGAGCAAATGAAAAAG
p62-211-384-EcoRI-R	CCGGAATTCTCAAGCTTCCTTCAG
6×His/GST-p62-UBA-F-EcoRI	CCGGAATTCCTCCCGCCAGAG
6×His/GST-p62-UBA-R-XhoI	CCGCTCGAGTCACAACGGCGG

p62-K420R-F	CTACAGACCAGGAATTACGAC
p62-K420R-R	GTCGTAATTCCTGGTCTGTAG
p62-K420Q-F	CTACAGACCCAGAATTACGAC
p62-K420Q-R	GTCGTAATTCTGGGTCTGTAG
p62-K435R-F	CAGTATTCGAGGCACCCTCCA
p62-K435R-R	TGGAGGGTGCCTCGAATACTG
p62-K435Q-F	CAGTATTCGCAGCACCCTCCA
p62-K435Q-R	TGGAGGGTGCTGCGAATACTG
p62-D69A-F	CACTACCGCGCAGAGGATGGG
p62-D69A-R	CCCATCCTCTGCGCGGTAGTG
p62-K7A-F	TTCACGGTGGCGGCCTATCTT
p62-K7A-F	AAGATAGGCCGCCACCGTGAA
pEP-*p62*-KO-F	ACCGTGAATTTCCTGAAGAACGT
pEP-*p62*-KO-R	AACACGTTCTTCAGGAAATTCAC
HDAC6-H216A-F	ATTAGGCCTCCTGGACATGCAGCCCAGCACAGT
HDAC6-H216A-R	ACTGTGCTGGGCTGCATGTCCAGGAGGCCTAAT
HDAC6-H611A-F	CAGGACACGCAGCAGAGCAGGAT
HDAC6-H611A-R	ATCCTGCTCTGCTGCGTGTCCTG
TIP60-Q377/G380E-F	CTCCCTACGAACGCCGGGAATACCGGAAG
TIP60-Q377/G380E-R	CTTCCGGTATTCCCGGCGTTCGTAGGGAG
p62-mLIR-F	AGACGATGACGCAACACATGCATCTTCAAAAG
p62-mLIR-R	CTTTTGAAGATGCATGTGTTGCGTCATCGTCT

Supplementary Table 2 List of shRNAs used in this study.

shRNAs	Sequences (5'-3')
shNC-F	GATCCGAAGGGATGGCAGAGAAGCTTCAAGAGAGCTTCTCTGCCATCCCTTCTTTTTTG
shNC-R	AATTCAAAAAAGAAGGGATGGCAGAGAAGCTCTCTTGAAGCTTCTCTGCCATCCCTTCG
sh*p300*-F	GATCCGCCCGGTGAACTCTCCTATAATTTCAAGAGAATTATAGGAGAGTTCACCGGGCTTTTTTG
sh*p300*-R	AATTCAAAAAAGCCCGGTGAACTCTCCTATAATTCTCTTGAAATTATAGGAGAGTTCACCGGGCG
sh*PCAF*-F	GATCCGCAGATACCAAACAAGTTTATTTCAAGAGAATAAACTTGTTTGGTATCTGCTTTTTTG
sh*PCAF*-R	AATTCAAAAAAGCAGATACCAAACAAGTTTATTCTCTTGAAATAAACTTGTTTGGTATCTGCG
sh*GCN5*-F	GATCCGCTGAACTTTGTGCAGTACAATTCAAGAGATTGTACTGCACAAAGTTCAGCTTTTTTG
sh*GCN5*-R	AATTCAAAAAAGCTGAACTTTGTGCAGTACAATCTCTTGAATTGTACTGCACAAAGTTCAGCG
sh*TIP60*-1-F	GATCCGTCGAATTGTTTGGGCACTGATTTCAAGAGAATCAGTGCCCAAACAATTCGACTTTTTTG
sh*TIP60*-1-R	AATTCAAAAAAGTCGAATTGTTTGGGCACTGATTCTCTTGAAATCAGTGCCCAAACAATTCGACG
sh*TIP60*-2-F	GATCCGCCTCAATCTCATCAACTACTATTCAAGAGATAGTAGTTGATGAGATTGAGGCTTTTTTG
sh*TIP60*-2-R	AATTCAAAAAAGCCTCAATCTCATCAACTACTATCTCTTGAATAGTAGTTGATGAGATTGAGGCG
sh*TIP60*-3-F	GATCCGCCTCCTATCCTATCGAAGCTATTCAAGAGATAGCTTCGATAGGATAGGAGGCTTTTTTG
sh*TIP60*-3-R	AATTCAAAAAAGCCTCCTATCCTATCGAAGCTATCTCTTGAATAGCTTCGATAGGATAGGAGGCG
sh*HDAC6*-F	GATCCGCTGCAAGGGATGGATCTGAACTTCAAGAGAGTTCAGATCCATCCCTTGCAGCTTTTTTG
sh*HDAC6*-R	AATTCAAAAAAGCTGCAAGGGATGGATCTGAACTCTCTTGAAGTTCAGATCCATCCCTTGCAGCG
