# Supplementary material for: Requirement for p62 acetylation in the aggregation of ubiquitylated proteins under nutrient stress
Source: Nat Commun. 2019 Dec 19;10:5792. doi: 10.1038/s41467-019-13718-w (PMC6923396; doi:10.1038/s41467-019-13718-w)
Supplement: Supplementary file 3 — Reporting Summary [file 41467_2019_13718_MOESM3_ESM.pdf]

nature research

Corresponding author(s): Wei Liu

Last updated by author(s): Nov 11, 2019

Reporting Summary

Nature Research wishes to improve the reproducibility of the work that we publish. This form provides structure for consistency and transparency in reporting. For further information on Nature Research policies, see Authors & Referees and the Editorial Policy Checklist.

Statistics

For all statistical analyses, confirm that the following items are present in the figure legend, table legend, main text, or Methods section.

n/a	Confirmed	
☐	☒	The exact sample size (*n*) for each experimental group/condition, given as a discrete number and unit of measurement
☐	☒	A statement on whether measurements were taken from distinct samples or whether the same sample was measured repeatedly
☐	☒	The statistical test(s) used AND whether they are one- or two-sided<br>*Only common tests should be described solely by name; describe more complex techniques in the Methods section.*
☒	☐	A description of all covariates tested
☐	☒	A description of any assumptions or corrections, such as tests of normality and adjustment for multiple comparisons
☐	☒	A full description of the statistical parameters including central tendency (e.g. means) or other basic estimates (e.g. regression coefficient) AND variation (e.g. standard deviation) or associated estimates of uncertainty (e.g. confidence intervals)
☒	☐	For null hypothesis testing, the test statistic (e.g. *F*, *t*, *r*) with confidence intervals, effect sizes, degrees of freedom and *P* value noted<br>*Give P values as exact values whenever suitable.*
☒	☐	For Bayesian analysis, information on the choice of priors and Markov chain Monte Carlo settings
☒	☐	For hierarchical and complex designs, identification of the appropriate level for tests and full reporting of outcomes
☒	☐	Estimates of effect sizes (e.g. Cohen's *d*, Pearson's *r*), indicating how they were calculated

Our web collection on statistics for biologists contains articles on many of the points above.

Software and code

Policy information about availability of computer code

Data collection	Zeiss LSM 800 confocal microscope; Bruker ASCEND 600MHz NMR magnet system; Odyssey infrared imaging system_v3.0; SpectraMax M5/M5e.
Data analysis	Zen Lite 2012; Microsoft Excel 2007; NMRPipe_v2010; CCPN Analysis_v2.4.2; GraphPad Prism 8.0; Image J_v1.8.0; PyMOL_v2.3.2.

For manuscripts utilizing custom algorithms or software that are central to the research but not yet described in published literature, software must be made available to editors/reviewers. We strongly encourage code deposition in a community repository (e.g. GitHub). See the Nature Research guidelines for submitting code & software for further information.

Data

Policy information about availability of data

All manuscripts must include a data availability statement. This statement should provide the following information, where applicable:
- Accession codes, unique identifiers, or web links for publicly available datasets
- A list of figures that have associated raw data
- A description of any restrictions on data availability

The data that support this study are available from the corresponding authors upon reasonable request.

Field-specific reporting

Please select the one below that is the best fit for your research. If you are not sure, read the appropriate sections before making your selection.

☒ Life sciences ☐ Behavioural & social sciences ☐ Ecological, evolutionary & environmental sciences

Life sciences study design

All studies must disclose on these points even when the disclosure is negative.

Sample size	Sample sizes were determined depending on the experimental settings.
Data exclusions	No data was excluded.
Replication	Unless specified, experiments were confirmed with three biological replicates, and the representative results were shown.
Randomization	No randomization was adopted in this study.
Blinding	No blinding was adopted in this study.

Reporting for specific materials, systems and methods

We require information from authors about some types of materials, experimental systems and methods used in many studies. Here, indicate whether each material, system or method listed is relevant to your study. If you are not sure if a list item applies to your research, read the appropriate section before selecting a response.

Materials & experimental systems

n/a	Involved in the study	
☐	☒	Antibodies
☐	☒	Eukaryotic cell lines
☒	☐	Palaeontology
☒	☐	Animals and other organisms
☒	☐	Human research participants
☒	☐	Clinical data

Methods

n/a	Involved in the study	
☒	☐	ChIP-seq
☒	☐	Flow cytometry
☒	☐	MRI-based neuroimaging

Antibodies

Antibodies used

Primary antibodies:

Rabbit anti-ATG7 (A2856, Sigma-Aldrich), 1:1000 dilution for western blot;
Rabbit anti-α-tubulin (Lys40) (SAB5600134, Sigma-Aldrich), 1:1000 dilution for western blot;
Mouse anti-β-actin (A5316, Sigma-Aldrich), 1:3000 dilution for western blot;
Mouse anti-α-tubulin (T5293, Sigma-Aldrich), 1:3000 dilution for western blot;
Rabbit anti-Flag (0912-1, HuaBio), 1:1000 dilution for western blot, 1:100 for immunoprecipitation and immunofluorescence;
Rabbit anti-HA (0906-1; HuaBio), 1:1000 dilution for western blot, 1:100 for immunoprecipitation;
Rabbit anti-p300 (SC-585, Santa Cruz); 1:200 dilution for western blot;
Rabbit anti-GST (SC-33613, Santa Cruz); 1:500 dilution for western blot;
Mouse anti-Flag (SC-807, Santa Cruz); 1:500 dilution for western blot, 1:100 for immunoprecipitation;
Mouse anti-HA (SC-57592, Santa Cruz); 1:500 dilution for western blot, 1:100 for immunoprecipitation;
Mouse anti-acetylated-lysine (SC-32268, Santa Cruz); 1:500 dilution for western blot, 1:100 for immunoprecipitation;
Mouse anti-GCN5 (SC-365321; Santa Cruz); 1:200 dilution for western blot;
Mouse anti-HDAC6 (SC-28386, Santa Cruz); 1:200 dilution for western blot;
Mouse anti-ubiquitin (SC-8017; Santa Cruz); 1:500 dilution for western blot, 1:100 for immunofluorescence;
Rabbit anti-GFP (598, MBL); 1:1000 dilution for western blot;
Rabbit anti-PCAF (C14G9, Cell Signaling Technology); 1:1000 dilution for western blot, ;
Rabbit anti-acetylated-lysine (9441, Cell Signaling Technology); 1:1000 dilution for western blot; 1:100 for immunoprecipitation;
Rabbit anti-phospho-tyrosine (9411, Cell Signaling Technology); 1:1000 dilution for western blot;
Rabbit anti-TIP60 (10827-1-AP; Proteintech); 1:1000 dilution for western blot;
Rabbit anti-p62 (18420-1-AP, Proteintech); 1:1000 dilution for western blot, 1:100 for immunoprecipitation;
Rabbit anti-phospho-serine (61-8100, Invitrogen); 1:1000 dilution for western blot.

Secondary antibodies:

Donkey anti-mouse IRDye680 (926-32222; LI-COR Biosciences); 1:3000 dilution for immunofluorescence;
Donkey anti-rabbit IRDye800CW (926-32212; LI-COR Biosciences); 1:3000 dilution for immunofluorescence;
Donkey anti-rabbit Alexa Fluor 488 (A21206, Invitrogen); 1:300 dilution for immunofluorescence;
Goat anti-mouse Alexa Fluor 635 (A31574, Invitrogen); 1:300 dilution for immunofluorescence.

Validation

All the antibodies have been verified by the manufacturers according to the introductions on their websites.

Eukaryotic cell lines

Policy information about cell lines

Cell line source(s)	HEK293, HEK293T and HeLa cells were obtained from ATCC (http://www.atcc.org/). p62-KO HEK293 cells were generated by using CRISPR-Cas9 system.
Authentication	HEK293, HEK293T and HeLa cells used in this study were verified by ATCC (http://www.atcc.org/). p62-KO HEK293 cells were verified by western blot and immunofluorescence.
Mycoplasma contamination	The cell lines were not tested for mycoplasma contamination.
Commonly misidentified lines (See ICLAC register)	There are on misidentified cell lines in this study.
